# Does a functional prosection provide a more effective method of learning the anatomy of the forearm and hand than a 3D online anatomy resource?

**DOI:** 10.15694/mep.2018.0000208.1

**Published:** 2018-09-12

**Authors:** Michael JH Smith, Tracey Wilkinson

**Affiliations:** 1 University of Dundee

**Keywords:** Anatomy, anatomy learning, prosection, 3D online resource, anatomy quiz, crossover study, medical students, medical school teaching, cadaver, Thiel embalming, prosection versus online anatomy resource, functional anatomy

## Abstract

This article was migrated. The article was marked as recommended.

Recent changes to anatomy education across UK medical schools are thought to be contributing towards a lower proficiency in anatomical expertise amongst students. The introduction of alternative learning methods may help to overcome this. Prosections and 3D online anatomy resources are both used as alternative methods for learning anatomy, but it is not clear which of these methods provides a better outcome.

The aim of this study was to compare students’ learning of the anatomy of the hand and forearm using a functional Thiel prosection or a 3D online resource to see which method was associated with a better outcome on an anatomy quiz. The secondary aim was to see which of these methods was preferred by students.

A cohort of 37 medical students at the University of Dundee participated in this crossover study. Group A learnt about the anterior compartment of the hand and forearm using the prosection followed by taking an appropriate quiz. They then used the 3D online resource to learn about the posterior compartment before again completing a relevant quiz. Group B carried out the study in reverse, using the prosection to learn about the posterior compartment and the 3D online resource to learn about the anterior compartment, each followed by completing the relevant quiz. All participants then completed a questionnaire about each of the learning methods.

The results showed no significant difference in quiz performance after using the Thiel prosection compared to using the 3D online resource (p>0.05). Feedback from questionnaires suggested that the majority of participants preferred using the prosection to learn functional anatomy.

Limitations of this study include the small sample size and the type of assessment method used. The results of this study were inconclusive and further studies are required to determine which resource is a better tool for learning anatomy.

## Introduction

Human anatomy is regarded as a fundamental and integral part of any medical education course ( Turney 2007; Papa et al. 2013) but the amount of anatomy teaching in medical schools has been in decline for a number of years ( McKeown et al. 2003).

The ‘traditional’ medical curriculum, which typically involved several years of basic science and anatomy teaching prior to clinical experience, has largely been replaced by a more integrated teaching format, including PBL/CBL (Problem/Cased-Based Learning) ( Gogalniceanu et al. 2009). Gross anatomy may no longer be taught as an entity in its own right, but is instead incorporated into basic science topics ( Turney 2007; Papa et al. 2013). Cadaveric dissection is consequently being used less across medical schools in the UK ( Dissabandara et al. 2015). This is partly due to a reduction in the number of cadavers nationally and the logistical difficulties of providing dissection teaching, but also that it is a less viable teaching option in this faster-paced, more clinically-driven curriculum ( Dissabandara et al. 2015).

Such changes to anatomy teaching seem not without fault; there was an increase in the number of claims associated with ‘anatomical error’ to the Medical Defence Union between 1995 and 2000 ( Dissabandara et al. 2015) and some surgeons believe that new medical school graduates possess less anatomical knowledge compared to previous students who were taught primarily through dissection ( Cottam 1999). Ghosh (2016) goes further and claims that dissection must be reinstated as the core method of learning anatomy to ensure safe medical practice.

There seems to be a need for an alternative method for learning anatomy that is more appropriate for the new curriculum formats than cadaveric dissection, but which provides the same level of learning opportunity. Two such methods, which are both already currently in use, are 3D online learning resources and prosections.

3D online resources are computer programmes that show the anatomy of the human body as high-definition computer generated images, allowing users to view specific regions or structures in isolation; they are accessible anytime and anywhere ( Singh et al. 2013; Ali et al. 2015; Iqbal 2016). Studies have suggested that students find such resources easy to use and helpful in understanding material to a higher level than learning from lectures alone ( Marsh et al. 2008), particularly when studying areas of anatomy which are otherwise difficult to visualise ( Nicholson et al. 2006). A further advantage is that such resources allow for standardisation of anatomy learning experiences on a global scale ( Brenton et al. 2007).

A study on the use of 3D online anatomy resources by Garg et al. (2001) found that the ability to view a 3D model with 360º rotation allowed participants to perform statistically better on an anatomy quiz (p<0.05). A similar study by Ruisoto et al. (2012) showed that participants using a 3D online model performed better in an associated quiz than participants who used 2D cross-sectional images. However, in comparing 3D online resources with dissection to learn forearm anatomy, Codd et al. (2011) found that there was no statistical difference (p>0.05) in quiz scores between two groups who used either dissection or an online resource.

Patel et al. (2015) explained that in the modern era, online resources are both more accessible and relevant to students who have grown up surrounded by technology. The authors went on to state that it was beneficial that online resources gave students greater autonomy over their studying, a belief which is contradicted by Levinson et al. (2007) who instead found that this autonomy led to users skipping sections of the resources or not spending sufficient time on them. Levinson et al. (2007) also discussed the importance of matching groups by gender when testing visual-spatial ability, as males performed significantly better than females on a quiz after using a 3D online anatomy resource, a finding supported by Codd et al. (2011).

In contrast to 3D online resources, prosections are physical specimens that maintain the spatial arrangement of structures found during dissections ( Ali et al. 2015) and allow users to interact in a ‘hands-on’ approach. Topp (2004) highlighted the ability to demonstrate normal anatomical variation as an advantage of prosections, also stating that they allow users to identify structures more quickly and easily compared to cadaveric dissection. An older study by Nnodim (1990) found a similar result; in this study, the prosection group both performed significantly better than the dissection cohort on a quiz (p<0.05) and completed it 26% quicker.

Different methods of embalming prosections can make them more suitable for various styles of teaching ( Cottam 1999). In Thiel embalming, tissues remain soft and joints are mobile ( Balta et al. 2014), making these prosections preferable over other learning methods for demonstrating the functional action of muscles ( Papa et al. 2013; Eisma et al. 2014). Kennel et al. (2017)compared student attitudes to dissection using Thiel and formalin embalmed cadavers and found that students using Thiel embalmed specimens were significantly more confident about their ability to recognise structures and to explore functional anatomy actively than those using formalin embalmed specimens. However, this preference for Thiel embalming was not matched with an improvement in anatomy knowledge on testing.

Plastination of human specimens produces durable models which have been said to allow users to appreciate areas of anatomy that are harder to visualise using dissection ( Papa et al. 2013), with over 90% of medical students at the University of Warwick agreeing that the use of such prosections was beneficial to their anatomy education ( Fruhstorfer et al. 2011).Conversely, Patel et al. (2008) found that professional anatomists still preferred using dissection over both prosections and 3D online resources for learning anatomy, a finding also supported by Kerby et al. (2010), who investigated medical student opinion.

The present literature thus suggests that both prosections and 3D online resources have inherent advantages and disadvantages and could be implemented in medical school curricula, but there is a lack of studies which directly compare these two methods. Therefore, the aims of this study were:

• to compare performance of students in an anatomy quiz after using a functional Thiel prosection or a 3D online anatomy resource
• to assess students’ attitudes towards each learning method

The hypotheses of the study were that students perform better in an anatomy quiz after using the Thiel prosection to learn the anatomy of the forearm and hand compared to an online resource, and that students prefer using the prosection over the online resource.

## Methods

**Figure 1.**
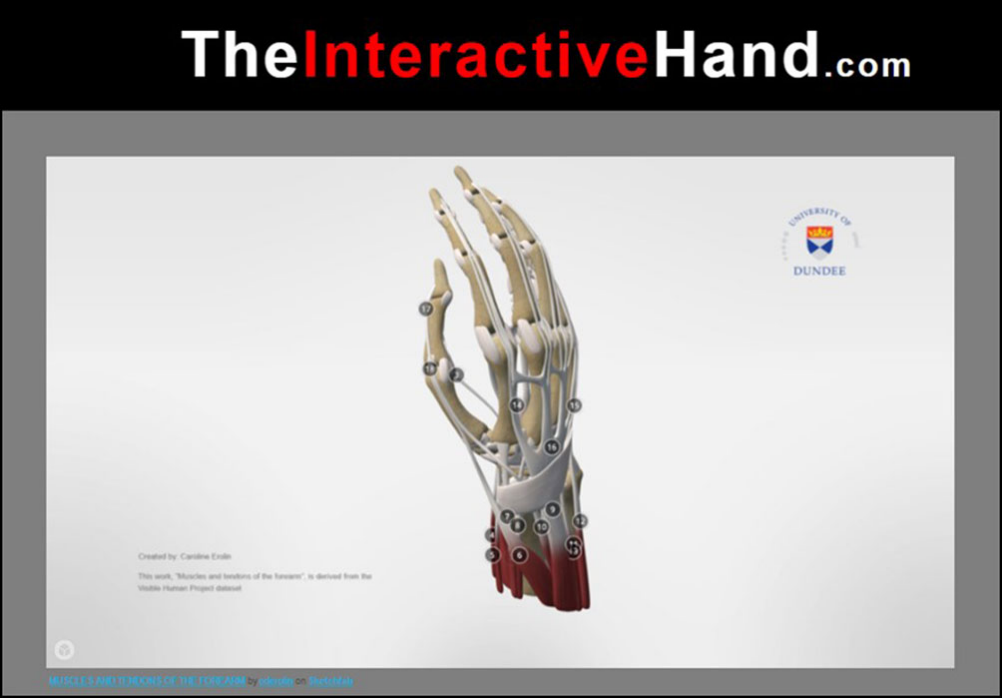
A still image from “The Interactive Hand”. This model shows the muscles and tendons of the forearm (adapted from Erolin 2016).

**Figure 2.**
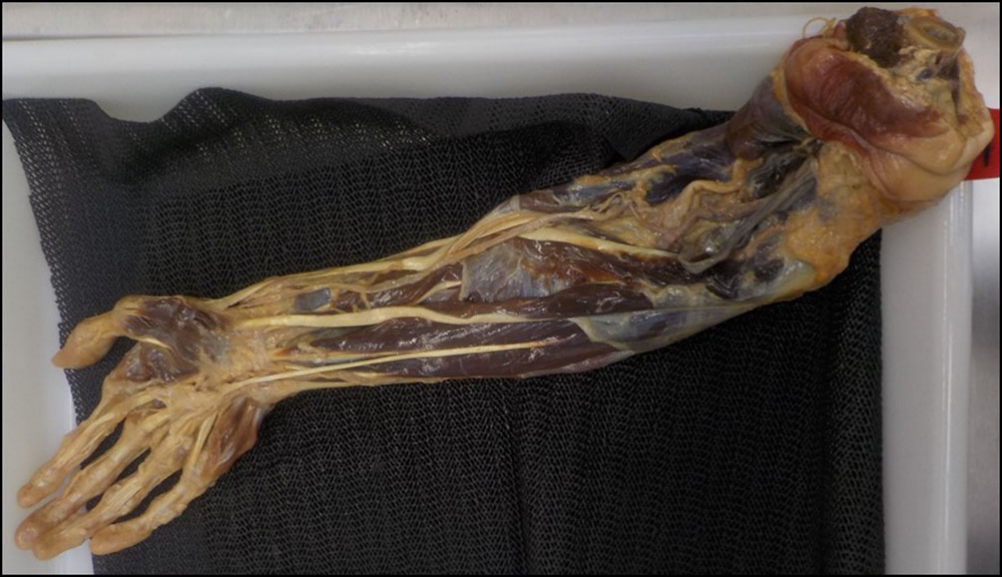
A view of the anterior forearm and hand of the prosection. Muscles are clearly visible due to removal of fascia.

The materials for this study consisted of an interactive 3D online learning resource of the right hand and forearm ( Erolin 2016) ( Figure 1) and a Thiel prosection showing the same structures ( Figure 2); the latter was used together with Clinical Anatomy Flashcards ( Gould 2008).

Ethical approval for the study was granted by the University of Dundee CAHID Ethics Committee.

Participants were recruited from Year 3 and Year 4 medical students at the University of Dundee but excluded students who had completed an anatomy degree previously. Recruited students were divided into 2 groups by allocating the individual into either Group A or Group B as they volunteered.

The project was carried out in the format of a crossover study ( Table 1). Group A learnt about the anterior compartment using the prosection and then undertook an appropriate quiz. They then used the 3D online learning resource to learn about the posterior compartment before again completing a relevant quiz. Group B carried out the study in reverse, using the prosection to learn about the posterior compartment and the 3D resource to learn about the anterior compartment.

**Table 1.** Crossover format of the study, in the ‘AB/BA’ style.

	LEARNING METHOD	
**ANATOMICAL AREA**	PROSECTION	3D ONLINE RESOURCE
ANTERIOR COMPARTMENT	GROUP A	GROUP B
	CROSSOVER	
POSTERIOR COMPARTMENT	GROUP B	GROUP A

Each participant used the learning resource for 25 minutes and was guided by a learning checklist for either the anterior or posterior compartment. Participants worked individually when using the 3D online learning resource and in groups of 1-5 when using the prosection. After using the resource, the participants had 20 minutes to complete the appropriate quiz individually. At the end of their second session, each participant anonymously completed a questionnaire.

The anterior and posterior compartment quizzes were each marked out of 20. The questions were a mixture of multiple choice and spot-style questions, with images used being a mixture of prosections and illustrations.

The questionnaire was designed to provide both quantitative and qualitative feedback. Section 1 was a Likert scale asking participants to respond to 20 statements. Sections 2, 3 and 4 of the questionnaire asked open questions about both learning methods.

### Data Analysis

Interpretation of the quantitative data took the form of 3 investigations, listed below. For all investigations, differences between the groups were tested with either a dependent or independent t-test or a Mann-Whitney U test and the level of significance used was p<0.05.

*Investigation 1:* To compare the level of difficulty between the two tests.

*Investigation 2:* To check that the two groups were of similar ability.

*Investigation 3:* To test whether there was a significant difference between quiz scores achieved after learning with the two resources.

## Results/Analysis

Thirty-seven participants took part in the study, with 18 in Group A, 9 of whom were from third year, and 19 participants in Group B, of whom 10 were from third year.

### Quizzes

*Investigation 1:* There was a statistically significant difference between the mean scores of the anterior compartment quiz (12.7±3.4) and posterior compartment quiz (14.7±2.7) (t=3.28; p=0.002).

*Investigation 2:* There was no difference between the mean score for participants in Group A (13.4±2.6) compared with Group B (14.0±3.6) (Mann-Whitney U=596.00; p=0.338).

*Investigation 3:* There was no difference between the mean scores of quizzes completed after using the prosection (14.3±3.1) or 3D online resource (13.1±3.2) (Mann-Whitney U=547.50; p=0.136).

### Questionnaire

Table 2 shows the results from Section 1 of the questionnaire. The statements are ranked in order from highest to lowest according to the number of participants who agreed with each statement.

**Table 2.** Results from Section 1 of the questionnaire.

Question Number	Statement	Agree	Neither Agree nor Disagree	Disagree
**16**	I enjoyed being able to act out muscle actions using the prosection	34	1	2
**1**	I enjoyed the interactive aspect of the prosection	33	3	1
**13**	I would like to use the functional prosection to learn anatomy in the future	32	4	1
**7**	The prosection helped me to appreciate relationships between structures	32	3	2
**6**	The checklist provided enough guidance on what to study	29	5	3
**17**	The prosection made it easier for me to answer the spot questions involving pictures of other prosections	28	2	7
**2**	The 3D online resource was easy to use	27	7	3
**19**	The functional prosection was a better method of learning anatomy than the 3D online resource	21	11	5
**20**	Full body dissection is a better way of learning anatomy than using the 3D online resource	17	7	13
**12**	The 3D online resource helped me to visualise the movements of muscles	12	3	22
**18**	I preferred using the 3D online resource	10	8	19
**15**	The 3D online resource was confusing to use	10	4	23
**14**	I would prefer to learn anatomy using full body dissection instead of functional prosections	8	13	16
**10**	I preferred the spot style questions to the multiple choice questions	5	12	20
**9**	The test questions were too difficult	5	12	20
**3**	The 3D online resource did not give a good level of detail	5	7	25
**4**	I did not like using the flash cards with the prosection	5	3	29
**8**	The functional aspect of the prosection did not aid my learning	3	6	28
**5**	Using the prosection made me feel uncomfortable	1	1	35
**11**	The functional prosection was not interactive enough	0	3	34

## Discussion

The hypothesis stating that participants would perform better after using the Thiel prosection compared to after the 3D online resource was not supported, suggesting that neither resource was more effective than the other. Other reasons for this could be that the low sample size did not give sufficient power to the study or could be how participants used the resources, particularly the group sizes for each session. Due to having only one prosection available, participants worked in groups of up to five when learning using the prosection but worked individually during the 3D online resource session. As highlighted by Levinson et al. (2007), autonomous learning is thought to be disadvantageous whilst Laughlin et al. (2006)suggested that working in small groups is beneficial to learning. This factor of working in groups versus individually was not accounted for and it is unknown how it may have influenced student performance. Future studies should aim to have participants in equal sized groups when using both learning methods. Further improvements with regard to grouping can be made by matching participants between each of the cohorts at the start of the study. There was no significant difference between the mean scores of the groups, which suggests that they were evenly matched. Studies have shown that matching participants by gender and visual-spatial ability can influence results ( Levinson et al. 2007; Codd et al. 2011) and this could be implemented in future studies.

The second hypothesis, that participants would prefer using the prosection versus the 3D online resource, was supported. In comparing using the Thiel prosection to the 3D online resource, over half of the participants agreed that: “The functional prosection was a better method of learning anatomy than the 3D online resource”. Many participants highlighted the interactive nature of the prosection as a positive feature, with 34 participants agreeing with the statement: “I enjoyed being able to act out muscle actions using the prosection”. The questionnaire results also showed that the majority of participants found this functional element of the prosection to improve their learning, with one participant writing: *“Prosection is better for visualising function”* and another: “ *Useful to test the movements*”. These results support the findings of Papa et al. (2013) and Eisma et al. (2014) in relation to the advantages of using Thiel embalming. This is in contrast to feedback on the 3D online resource, where the majority of participants disagreed with the statement: “The 3D online resource helped me to visualise the movements of muscles”.

Participant feedback regarding the learning methods could be influenced by the ease with which they completed the associated quiz. The quizzes were designed to contain spot-style questions featuring images of both cadaveric material and illustrations; 28 of 37 participants agreed with the statement: “The prosection made it easier for me to answer the spot questions involving pictures of other prosections”. If participants found the quiz easier to complete after using the prosection, then, regardless of actual performance, they may comment favourably on that associated learning method. Subsequent studies could investigate this idea further to establish if using a certain learning resource improves performance when answering questions which feature images or material from that same resource type.

Similar to Kennel et al. (2017),who found that student preference for Thiel embalmed cadavers was not matched by better anatomy knowledge, student preference for the use of the prosection over the 3D online resource was not reflected in an improved quiz performance after using the prosection. Limitations of the study could be responsible for this finding. Due to logistical difficulties, the exact order for which individuals completed the study was not specified. Therefore, the fact that participants scored significantly better on the posterior compartment quiz than anterior compartment quiz could be due to more individuals undertaking their anterior compartment session first, gaining insight into the study and developing skills which they implemented during their second session on the posterior compartment, thus performing better.

Further limitations include the small sample size of 37 participants and that the sample population may not be representative of the general medical student population, as individuals volunteered for this study and so may have a special interest in this topic. The robustness of the assessment method should also be questioned; participants had 25 minutes to use a learning resource immediately before completing the quiz. This format did not allow for testing of knowledge retention and the time-frame was insufficient to allow participants to become proficient at using each learning method. An improvement would be to develop a study with a strict order for undertaking learning and assessment sessions which is integrated with the curriculum; this would increase sample size, provide a more representative sample cohort and examine the long-term effects of using different learning methods.

It was suggested from the results of the questionnaire that although participants clearly preferred the prosection to the 3D online resource, there was a less definitive preference when comparing each learning method to cadaveric dissection. Seventeen out of 37 participants agreed that: “Full body dissection is a better way of learning anatomy than using the 3D online resource” whereas 8 participants agreed with, and 13 individuals were neutral to, the statement: “I would prefer to learn anatomy using full body dissection instead of functional prosections”. This result is in contrast to findings by both Patel et al. (2008) and Kerby et al. (2010)who found a clear preference for dissection over other methods. This finding is particularly interesting; medical students at the University of Dundee are taught anatomy primarily through dissection and prosection, which is the same teaching method implemented at both Imperial College London and the University of Nottingham, where Kerby et al. (2010) conducted their study. This difference in opinion between medical students who are fundamentally taught using the same learning modality could indicate that it is not so much the learning modality that is important but the way it is integrated into the curriculum. Further investigation into differences between course structures and delivery of anatomy teaching would be required to understand this finding fully. Ghosh (2016)advocated the use of dissection but conceded that it is not suitable as a sole learning method and instead recommended that dissection is complemented by other interactive learning methods. Therefore, a future study should aim to include an additional cohort which uses dissection as a learning method to allow for a 3-way comparison and to examine if a combination of such learning methods provides a more advantageous learning experience.

## Conclusion

This study has shown that students perform equally well on an anatomy quiz after using either a Thiel prosection or a 3D online resource as learning methods. This result does not support the hypothesis that use of a prosection rather than a 3D online resource leads to improved performance.

Feedback from the questionnaire showed that students preferred to use a prosection compared to a 3D online resource, supporting the second hypothesis.

Further studies are required to assess fully the effectiveness of both these learning methods at helping students to study anatomy.

## Take Home Messages

Although medical students may prefer to study anatomy using a functional prosection, this learning method does not appear to have a significant effect on their performance in anatomy quizzes compared to use of a 3D online resource.

## Notes On Contributors

Michael Smith is a 5th year medical student at the University of Dundee, Scotland, UK. This study was completed as part of his intercalated BMSc degree in anatomy.

Tracey Wilkinson is the Cox Chair of Anatomy at the University of Dundee, Scotland, UK.
