# Supplementary material for: Does a functional prosection provide a more effective method of learning the anatomy of the forearm and hand than a 3D online anatomy resource?
Source: MedEdPublish (2016). 2018 Sep 12;7:208. [Version 1] doi: 10.15694/mep.2018.0000208.1 (PMC10701834; doi:10.15694/mep.2018.0000208.1)
Supplement: Peer review history

# Reviewer report: Reviewer response for version 1

**DOI:** 10.21956/mep.19664.r29355

**Published:** 2018-10-09

**Authors:** Cindy Ahearn

**Affiliations:** 1 The University of Melbourne

This review has been migrated. The reviewer awarded 4 stars out of 5 9/10/18Title: Does a functional prosection provide a more effective method of learning the anatomy of the forearm and hand than a 3D online anatomy resource?Dear Colleague, Firstly, thank you for submitting and allowing your work to be a part of the academic review process. Congratulations for getting your work to this point and bringing your work into public view. This feedback is sent with generosity of spirit and collegiality in the hopes of assisting your learning journey. Entitled “Does a functional prosection provide a more effective method of learning the anatomy of the forearm and hand than a 3D online anatomy resource?” The reviewer enjoyed the central idea in this paper: that changes in anatomy learning may lower student proficiencies. The genesis of this paper is that the introduction of alternative learning methods could assist the field and identifies a complexity as to best methods.The introduction is logical and well scaffold to present a clear academic through-line. The manuscript describes a general cohesion of contemporary education approaches to alternative anatomy learning, with efficiency. The reviewer likes the identification of visual learning methods versus lectures as purposeful. The importance of this paper stems from the applicability of the approach to teaching and learning as important; offers clear insight in terms of innovation might not always be best for economically challenged schools. Although this work presents a general paradigm that can be broadly and usefully applied, its balance of positive and negatives linked to relevant literature lifts its tendency toward middle ground. The manuscript is well-written and nicely presented, with a good balance of descriptive/visual text, discourse and practical solutions. Limitations are outlined clearly and demonstrates author understanding of research task. Some elements of the discussion [physical 3D results/computer generated] as results could have been connected to motor elements of learning, which slightly weakens the strength and potential of the paper. The main strength of this paper, however, is that it addresses an interesting repetition in teaching and learning and identifies a pragmatic solution. This reviewer would have like more fusion between results/analysis bridged for the discussion. An opportunity to illumine results insight would be useful before moving onto Discussion, which in this case is really the analysis. It is clear that this article will make a valuable contribution to method of learning in anatomy education and its neutral suggestion that students perform equally well using either method is therefore not distinctive. This is a solid example of a generally well-managed educational research effort. The reviewer wishes the authors a successful publication. Best of luck.

Reviewer Expertise:

NA

No decision status is available

# Reviewer report: Reviewer response for version 1

**DOI:** 10.21956/mep.19664.r29354

**Published:** 2018-09-12

**Authors:** Trevor Gibbs

**Affiliations:** 1 AMEE

This review has been migrated. The reviewer awarded 4 stars out of 5 An interesting paper written by a medical student. It is important during any research to make sure that the research under-taken is relevant, to look at the negative as much as the positive effects of the research and to be able to highlight clearly the limitations of the research undertaken. I feel that this paper covers all those areas. Introduction of new areas of teaching and learning is very important, especially in resource-restrained schools- being new and innovative often costs more and might not always be better.The authors clearly expressed the negative outcomes of their researchThe extensive section on limitations clearly showed that the author(s) thought deeply about their research. My only comment here would be some element of discussion of whether a physical 3D model would have given the same results as the computer generated 3D model- my instinct would suggest that it would due to the psycho-motor element of learning. A well written piece of research which although not going to change much in our learning is a very good example of educational research.

Reviewer Expertise:

NA

No decision status is available
